# Presence of Halogenated Polycyclic Aromatic Hydrocarbons in Milk Powder and the Consequence to Human Health

**DOI:** 10.3390/toxics10100621

**Published:** 2022-10-19

**Authors:** Prasun Goswami, Anura Upasanta-Kumara Wickrama-Arachchige, Momoka Yamada, Takeshi Ohura, Keerthi S. Guruge

**Affiliations:** 1 Atal Centre for Ocean Science and Technology for Islands, ESSO-National Institute of Ocean Technology, Port Blair 744103, Andaman and Nicobar Islands, India; 2 Faculty of Fisheries and Ocean Sciences, Ocean University of Sri Lanka, Tangalle HB 82200, Sri Lanka; 3 Faculty of Agriculture, Meijo University, Nagoya 468-8502, Japan; 4 National Institute of Animal Health, National Agriculture and Food Research Organization, Tsukuba 305-0856, Japan; 5 Graduate School of Life and Environmental Sciences, Osaka Metropolitan University, Osaka 598-8531, Japan

**Keywords:** halogenated polycyclic aromatic hydrocarbons, milk powder, Sri Lanka, Japan, Orbitrap GC/MS

## Abstract

Recent reports of the presence of halogenated derivatives of polycyclic aromatic hydrocarbons (PAHs) in human foods of animal origin, such as chlorinated (ClPAHs) and brominated (BrPAHs) PAHs, suggest that their contamination in dairy products may also pose a human health risk. This study used GC/Orbitrap-MS to analyze 75 congeners of halogenated PAHs and parent PAHs in milk and creaming powder samples commonly found in grocery stores in Sri Lanka and Japan. Our investigation revealed a total of 31 halogenated PAHs (HPAHs) in the samples. The concentrations of total parent PAHs in the samples from Sri Lanka and Japan ranged from not detected (n.d.)–0.13 and <0.001–16 ng/g dry weight (d.w.). Total ClPAHs and BrPAHs in the samples ranged from 0.01–3.35 and 1.20–5.15 ng/g (d.w.) for Sri Lanka, and 0.04–2.54 and n.d.–2.03 ng/g d.w. for Japan, respectively. The ClPAHs were dominated by chlorinated-pyrene, -fluoranthene, and -benzo[a]pyrene congeners, whereas the BrPAHs were dominated by brominated-naphthalene and -pyrene congeners. The toxic assessment estimated based on the intake of toxic equivalency quotients (TEQs) for target compounds in milk powders revealed that HPAHs might contribute additively to the PAHs-associated health risk to humans, indicating that more research is needed.

## 1. Introduction

Dairy products are possibly the most essential and irreplaceable food items in modern society for both infants and adults. Dairy product quality and safety are critical, especially for pediatric nutrition. It is important to reduce the risk of harmful compounds in milk products, including polycyclic aromatic hydrocarbons (PAHs), which are ubiquitous in the environment. Milk is thought to be contaminated with PAHs due to its fat content because PAHs are lipophilic [1]. Grazing on contaminated grass near industrialized areas, as well as the contaminated soil that is ingested with the grass, results in high levels of PAHs in ruminants’ milk [2,3,4]. Unlike PAHs, some of their halogenated PAH (HPAH) congeners are prevalent at one to two orders lower levels in diverse environmental matrices, including human food items. However, their mechanisms of genesis are thought to be similar [5,6,7,8]. Despite their low concentrations, many ClPAHs and BrPAHs congeners have toxic effects comparable to dioxins [9].

Structural similarities between HPAHs and toxic organochlorine compounds suggest that they may have similar toxic effects. Ohura et al. [9] reported that there was a relatively higher aryl hydrocarbon receptor activities in low molecular weight BrPAHs than that of corresponding PAHs. Horii et al. [10] found that certain HPAHs have dioxin-like toxicities. Furthermore, organ-and gender-specific acute toxic effects have been observed in ICR mice when the experiment was performed using 7-chloro-benzo[a]anthracene [11,12]. These findings suggest that HPAHs may pose health hazards potentially for humans, so the evaluation of concentrations in various foods, including milk and dairy products, and the assessment of human health risk is very important.

Previous studies have shown a wide range of PAHs and HPAHs contamination in various animal products (reviewed by Amirdivani et al. [13], Masuda et al. [5], Yan et al. [14]). We have recently reported concentrations of many ClPAHs and BrPAHs in edible aquatic species indicating that they have a high potential to bioaccumulate in the fish tissue [8,15]. Ding et al. [16] also reported the bioaccumulation of a few ClPAHs in Chinese rice crop samples. Similarly, PAHs and HPAHs have been linked to human risk from contaminated meat and seafood [5,8,15]. To the best of our knowledge, no study has yet confirmed the accumulation of more than 50 HPAH congeners in milk and other dairy products.

For baby foods and processed cereal-based foods for infants and young children, European Union (EU) regulations set a concentration limit of less than 1 mg/kg of benzo[a]pyrene (BaP) [17]; however, due to the lack of baseline data, no such limit has been set for the HPAHs. Nonetheless, the risk levels of PAHs and HPAHs for human exposure via diet and cooking exhaust gas have been evaluated using the BaP-like Toxic Equivalency Quotient (TEQ), and the cancer risk induced by HPAHs and PAHs in seafood, cooked food, and cooking exhaust gas, respectively [5,18]. Different fish species were investigated for HPAHs and PHAs in Sri Lanka and Japan, and the concentrations of HPAH congeners in the fish from Sri Lanka were approximately ten times higher than the corresponding concentrations in Japanese samples [15]. In addition, TEQ-estimated cancer risks and the intake of PAHs and HPAHs by consuming aquatic organisms showed that exposure levels in several species could exceed the acceptable risk level for Sri Lankans [15]. Thus the goals of this study were to (1) elucidate the congener-specific accumulation of PAHs, ClPAHs, and BrPAHs in various types of milk powder available in Sri Lanka and Japan and (2) evaluate toxic outcomes due to consumption of tested milk products in which we used TEQs for several target compounds to calculate the cumulative toxic risk of PAHs and HPAHs. We used gas chromatography (GC) Orbitrap mass spectrometry (MS), which is a high-throughput high-resolution accurate analytical method for detecting micropollutants. We estimated a collective BaP-like TEQs for PAHs and HPAHs using the toxic equivalency factors (TEFs) of PAHs, and the relative potencies (REPs) that were estimated from yeast AhR activity of each congener in place of the TEF [19] of HPAHs [15]. The assessment of the health risk of consuming contaminated milk powders will be useful as a springboard in preparing dietary guidelines for the consumption of milk and dairy products.

## 2. Materials and Methods

### 2.1. Sample Collection

A total of 8 milk powder samples were obtained from Sri Lankan and Japanese grocery stores (4 samples each). Three of the milk powders in the Sri Lankan samples were imported (SL-1, SL-2, and SL-4), while one sample (SL-3) was produced locally. Three milk or creaming powder samples from Japan were for adult consumption (JP-1, JP-3, and JP-4), while the JP-2 sample was a milk-based infant formula food. The supplementary section contains additional information and the fat content of each sample (Table S1).

### 2.2. Chemicals

The compounds of interest were 30 ClPAH congeners, 21 BrPAH (Table S2) congeners, and 24 PAH congeners (Table S2). The ClPAH and BrPAH standards synthesized similar to the previous studies were used in this study [9]. The synthesized standards were stored at −85 °C. The 24 PAH congeners studied included 16 classified as priority pollutants by the U.S. Environmental Protection Agency. A multi-PAH mixed standard was procured from LGC Labor (Augsburg, Germany). Five deuterated PAHs (naphthalene-*d*_8_, phenanthrene-*d*_10_, pyrene-*d*_10_, benzo[a]pyrene-*d*_12_, and perylene-*d*_12_) were used as surrogate spikes, and fluoranthene-*d*_10_ was used as a syringe spike (Cambridge Isotope Laboratories, Tewksbury, MA, USA). All extraction and cleaning procedures were performed with analytical grade chemicals purchased from Wako Pure Chemical (Osaka, Japan) or Kanto Chemical (Tokyo, Japan).

### 2.3. Sample Extraction and Clean Up

A precise 2.0 g powder sample was weighed, and 50 μL of the above five deuterated PAHs (25 ng each) were added as a standard surrogate mixture. The extraction was then performed with an accelerated solvent extraction (ASE) system (Dionex ASE 350, Thermo Fisher Scientific, Waltham, MA, USA). The conditions for ASE extraction, silica gel cleaning, and gel permeation chromatography were reported in our previous study [8]. Under mild N₂ purge, the cleaned-up extract was concentrated to about 50 μL. Finally, a syringe spike of 50 μL of Fluoranthene-*d*_10_ (25 ng) and detection was performed using a high-resolution gas chromatography (GC) Orbitrap mass spectrometry (MS)-based method (Orbitrap GC/MS). A previous study described detailed Orbitrap GC/MS conditions for HPAHs and PAHs [8].

### 2.4. Quality Assurance and Quality Control

The recoveries of the surrogate standards spiked onto the samples were 7% ± 8% for naphthalene-*d*_8_, 26% ± 18% for phenanthrene-*d*_10_, 40% ± 12% for pyrene-*d*_10_, 101% ± 29% for benzo[a]pyrene-*d*_12_, and 93% ± 28% for perylene-*d*_12_. The recovery variations of internal standards have not influenced the quantification of target compounds that were performed using the isotope-dilution quantification method [8,15]. The target compounds concentrations detected in the procedural blank samples were subtracted before estimating concentrations in the real samples. The method detection limit (MDL) was 3 times the standard deviation of diluted measurable standard solutions. PAH and HPAH MDLs ranged from 0.25 (Py) to 25 pg (DBahP) and from 0.04 (9,10-Br2Ant) to 33 pg (Br3BaP), respectively.

### 2.5. Assessment of Potential Risks of Consuming Contaminated Milk Powder to Human Health

The intake risks for dioxin-like HPAHs and PAHs were calculated using toxic equivalents (TEQs), which are the sum of each congener’s concentrations weighted by the potency of its corresponding relative toxicity. The following equation gave the estimation:

(1) $$TEQ=\sum \left(PAH_{i}\times TEF_{i}\right)+\sum \left(ClPAH_{i}\times REP_{i}\right)+\sum \left(BrPAH_{i}\times REP_{i}\right)$$

where *i* denotes the *i*-th congener. It should be noted that the partial TEQs of PAHs were calculated using the toxic equivalency factor (TEF) based on the potency of BaP (TEF_BaP_ = 1). In contrast, the parts of HPAHs were calculated using the relative potency (REP) estimated from yeast AhR activity of each congener in place of the TEF [19]. Table S2 shows the TEF or REP value of each congener used in the assessment. Finally, the estimated TEQ values were multiplied by 2.6 to normalize by a unit teaspoon equivalent weight of milk powder (i.e., 2.6 g per serving) in dry weight.

### 2.6. Statistical Analysis

Before any statistical analysis, the concentration dataset was normalized using the log (x + 1) transformation. On the normalized data, two-way hierarchical clustering was performed with complete linkage and Euclidean distances as a measure of similarity. The heatmap was visualized using an online tool (www.heatmapper.ca accessed on 17 June 2021) and the method described by Babicki et al. [20]. STATISTICA (v. 10) was used to perform principal component analysis.

## 3. Results and Discussion

### 3.1. Concentrations of PAHs and HPAHs in Milk Powder Samples

**Figure 1:**
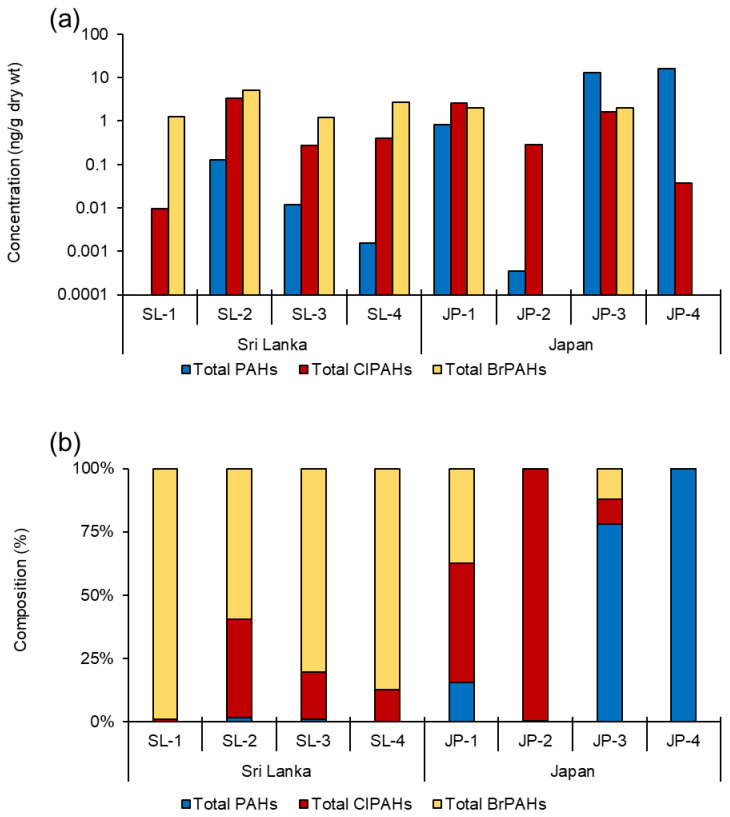
(**a**) Concentration of polycyclic aromatic hydrocarbons (PAHs), chlorinated polycyclic aromatic hydrocarbons (ClPAHs), and brominated polycyclic aromatic hydrocarbons (BrPAHs) (ng/g dry weight) in milk powder samples from Sri Lanka and Japan. (**b**) Composition of total PAHs, ClPAHs, and BrPAHs in milk powder samples from Sri Lanka and Japan.

To the best of our knowledge, this is the first attempt to study a wide range of ClPAHs and BrPAHs congeners in commercially available milk powder samples alongside their parent PAHs. A total of 75 target congeners were examined, with 53 detected in the samples. There was 20 parent PAHs detected out of 24 total, 23 ClPAHs detected out of 30 total, and 10 BrPAHs detected out of 21 total (Table S2). The concentrations of the total parent PAHs in milk powder samples from Sri Lanka and Japan ranged from not detected (n.d.)–0.13 and <0.001–16 ng/g dry weight (d.w.). Total ClPAHs and BrPAHs in the samples ranged from 0.01–3.35 and 1.20–5.15 ng/g (d.w.) for Sri Lanka, and 0.04–2.54 and n.d.–2.03 ng/g d.w. for Japan, respectively (Figure 1a). In the Sri Lankan milk powder samples, BrPAHs were the most prevalent, followed by ClPAHs, and very trace levels of parent PAHs were detected. Apart from the milk powder sample used for adult consumption, the signatures were significantly different in the Japanese milk powder samples, where ClPAHs and parent PAHs were most dominant (JP-1; Figure 1b). Milk powder is typically enriched with nutrient supplements using various spray-drying methods and preparation steps, particularly in formula feed [14]. The sole infant formula feed sample (JP-2) contained nine ClPAH congeners and a single congener of PAHs in the current study, but no BrPAHs were detected. The spatial differences in PAH levels in different milk and creaming powder samples indicated that the source of PAHs had a significant influence on PAHs contamination in biological samples, and these results are consistent with our previous findings in Sri Lankan and Japanese fish populations [15].

Total parent PAH contamination levels detected in Sri Lankan milk powder samples were nearly one to two orders of magnitude lower than levels reported from Argentina and Brazil [21], Italy [22], China [14], and Japan [3]; current study (Table 1). However, total PAHs levels detected in our Japanese samples were similar [14] or lower [21] than previously reported values, but at least one order higher than previously reported values of 0.47–1.32 ng/g of total PAHs from Romania [23]. When compared to the previous reports from Japan [3] or Italy [22], the total PAHs concentration in the only infant formula feed sample (JP-2) tested in this study was very low (<0.001 ng/g d.w.). The lack of previous studies on HPAHs accumulation in milk powder samples makes comparing our results difficult; however, in a recent study, Yan et al. [14] reported accumulation of 6-ClBaP congener up to 7.38 ng/g d.w. in infant milk powder samples from Shanghai, China. The maximum concentrations found in various adult and infant formulas in Shanghai were nearly twice as high as the values found in this study [14]. Because the BrPAHs levels in milk were not previously available, they could not be compared to current data.

### 3.2. Congener Differences in Milk Powder Samples

**Figure 2:**
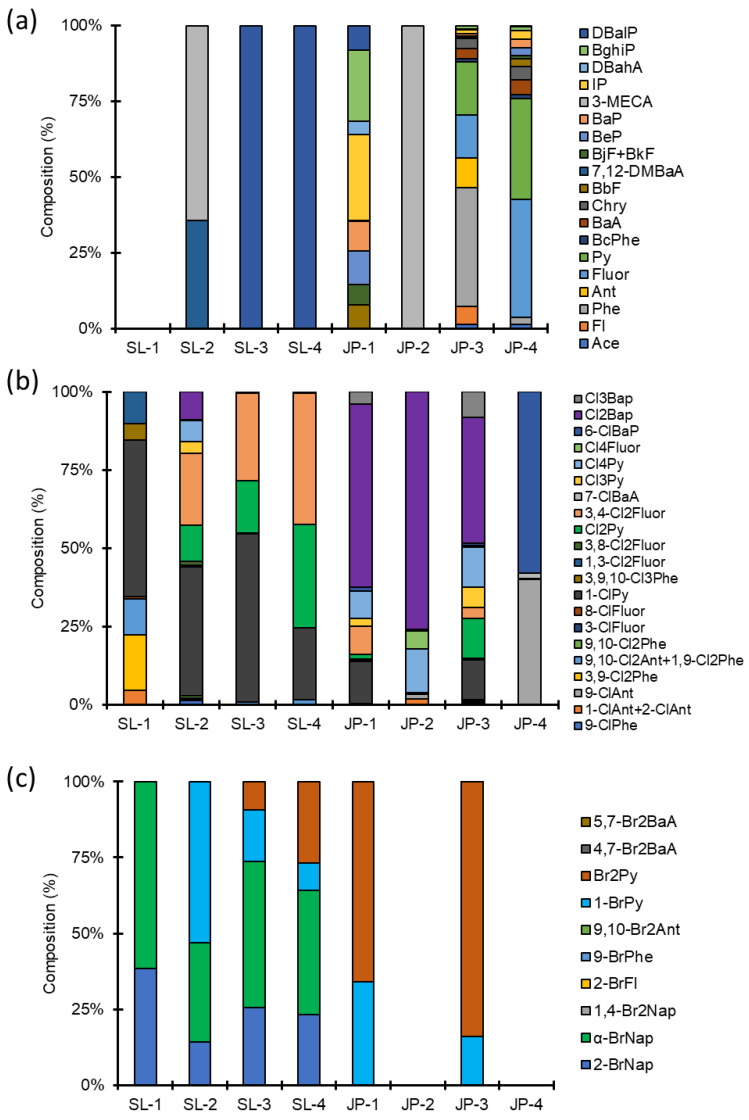
Congener-composition of (**a**) PAHs, (**b**) ClPAHs, and (**c**) BrPAH in milk powder samples from Sri Lanka and Japan.

When we compared different PAHs and HPAHs congeners’ profiles, we discovered a significant difference between Sri Lankan and Japanese milk powder samples (Figure 2). Very few parent PAH congeners were detected in Sri Lankan samples (Figure 2a). DBalP was the only detectable parent PAH congener in a locally produced milk powder sample (SL-3). However, in the imported milk powder samples (SL-1, SL-2, and SL-4), 3-MECA and 7, 12-DMBaA contributed up to 64% and 36%, respectively, for the SL-2 sample, while no parent PAHs were detected in the SL-1, but DBalP was detected in the SL-4. In Japanese milk powder samples, however, relatively high molecular weight PAH congeners (5–6 rings) were detected more frequently than low molecular weight (3–4 rings) PAH congeners (Table S2). In the JP-1, the three most dominant congeners were discovered to be IP (28%) > BghiP (24%) > BeP (11%). Other congeners such as BaP, DBalP, BbF, BbF + BjF, and DBahA contributed 4–10% of total parent PAHs. 3-MECA was the only parent PAH congener detected in the infant formula sample (JP-2). In the low-fat sample (JP-3), 17 different parent PAH congeners were detected. Phe (39%, 5.03 ng/g d.w.) > Py (17%, 2.25 ng/g d.w.) > Fluor (14%, 2.25 ng/g d.w.) were the three most dominant congeners. In JP-4, similar to JP-3, 17 different parent PAH congeners were detected. Fluor (39%, 6.25 ng/g d.w.) and Py (33%, 5.31 ng/g d.w.) contributed nearly 72% of total parent PAHs. These variations could be attributed to the various methods and ingredients used during the production process. PAHs are thought to be unintentionally produced during food processing and heating [5]. Moreover, further studies are required to demonstrate the relationship between lipid content and the accumulation of PAHs/HPAHs in the milk samples.

Among the ClPAHs, chlorinated pyrene, fluoranthene, and benzo[a]pyrene congeners were detected in all the samples (Figure 2b). Among these, the most prevalent congeners were 1-ClPy, Cl2Py, 3,4-Cl2Fluor, and Cl2BaP, indicating that these are the most persistent congeners in milk. A previous study on halogenated PAHs in sediment cores from Sri Lanka showed a relatively higher concentration of ClPAHs in surface layers, implying that incineration processes have likely caused ClPAH contamination in recent years [24]. Among the BrPAHs, 1-BrPy and Br2Py were the most predominant and were detected in both Sri Lankan and Japanese samples. Moreover, α-BrNap and 2-BrNap were in abundance in Sri Lankan samples but not in Japanese milk powders (Figure 2c). Wickrama-Arachchige et al. [15] also reported a predominance accumulation of chlorinated and brominated pyrenes in Sri Lankan aquatic samples, implying that halogenated pyrenes are more likely to be persistent biological matrices than others.

**Figure 3:**
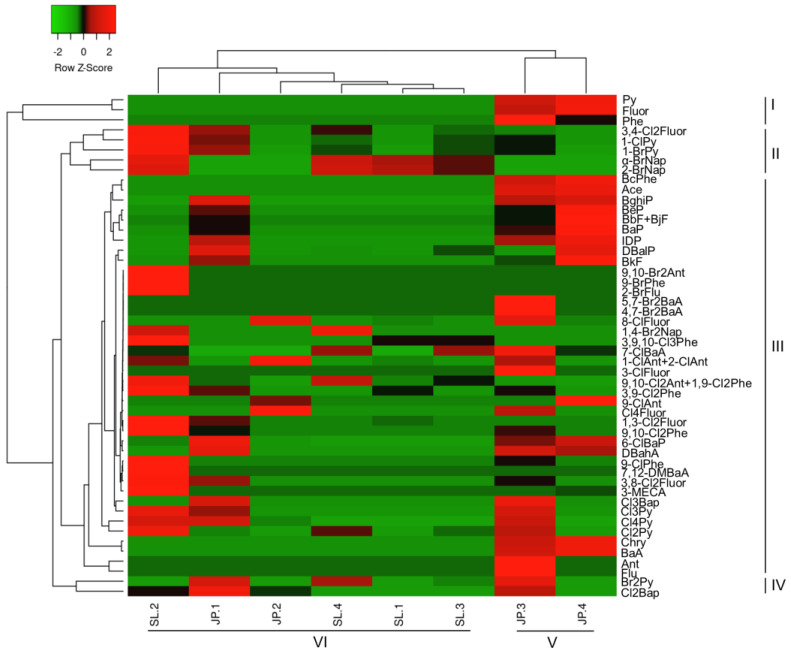
Cluster heat map showing the concentrations of detected PAHs, ClPAHs, and BrPAHs in milk powder samples from Sri Lanka and Japan. The relative concentrations of each congener in the milk powder samples are shown as intensities illustrated using the scales shown.

We used a hierarchical cluster-based heatmap and principal component analysis to understand the relationship between different PAH and HPAH congeners (Figure 3 and Figure S1A,B). For each congener, the heatmap produced three major clusters. Three parent PAHs, such as Py, Fluor, and Phe, were grouped in the first cluster (cluster I). These three compounds were detected in high concentrations in two Japanese milk powder samples (JP-3 and JP-4). The second cluster (cluster II) contained the most chlorinated, brominated, and parent PAHs, dominated mainly by halogenated pyrene, fluoranthene, phenanthrene, and anthracene. These results suggest that, despite geographic differences in the source regions of the samples, HPAHs are formed via similar processes to their parent congeners [9]. Individual clusters of Br2Py, Cl2BAP, 3, 4-Cl2Fluor, 1-ClPy, 1-BrPy, α-BrNap, and 2-BrNap were joined to form the third cluster. Because these congeners were found in higher concentrations at SL-2 and JP-1, their distribution patterns were similar. The clustering of different milk powder samples revealed two major clusters (clusters IV and V). The fourth cluster was formed by two JP 3 and JP4 milk creaming powder samples. These two samples had similar congener signatures when grouped together, which could have been due to their similar ingredients and processing; however, SL-1, SL-3, SL-4, and JP-2 formed a sub-cluster in the fifth cluster. Because the majority of the PAH concentrations in these samples were low, they were grouped. The second sub-cluster of cluster-V consists of SL-2 and JP-1, which are distantly related (Figure S1A,B). With a smaller sample size, it is difficult to predict the exact association between congeners or different samples; thus, a larger sample size is required for more in-depth analysis.

### 3.3. Exposure-Based Human Health Risk Assessment

**Figure 4:**
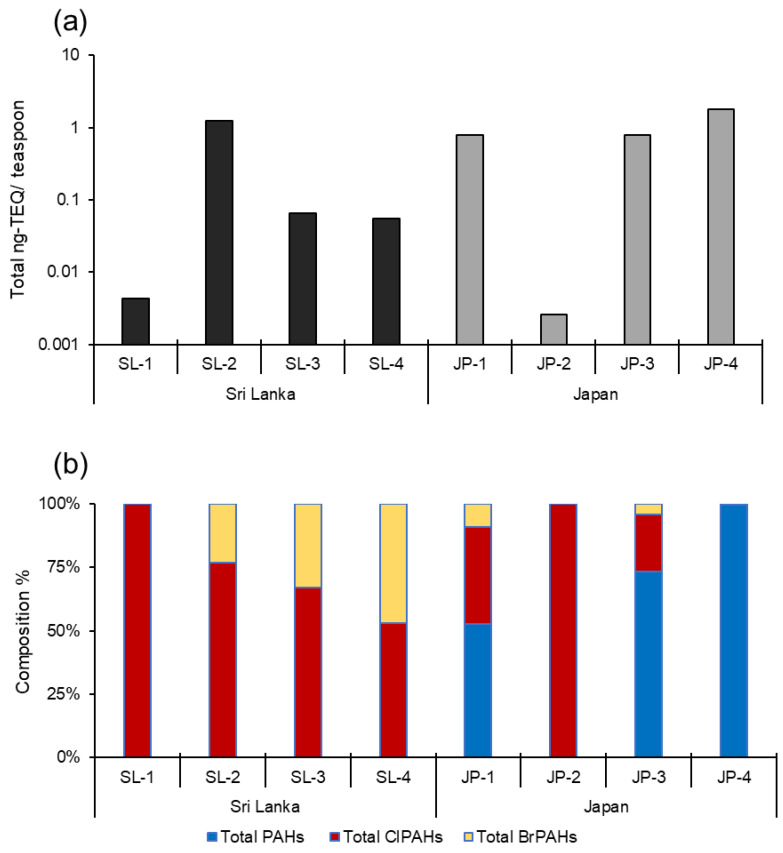
Dietary exposure-based toxic assessment calculated from toxic equivalent quotients (TEQ) for exposure to PAHs, ClPAHs, and BrPAHs. TEQ values were calculated for a teaspoon of milk powder in dry weight. (**a**) Total TEQ concentration and (**b**) composition of PAHs, ClPAHs, and BrPAHs.

TEQs were used to assess PAH and HPAH-mediated human health risks from these milk powder samples, which were normalized by a teaspoon equivalent weight of milk powder (i.e., 2.6 g per serving) in dry weight (Figure 4a, Table S3). JP-4 (creaming powder: 1.79 ng-TEQ/teaspoon) had the highest total TEQ (estimated with parent PAHs and HPAHs), while JP-2 (baby formula: 0.003 ng-TEQ/teaspoon) had the lowest. Three Sri Lankan samples, including the local product, had less than 0.07 ng-TEQ/teaspoon, eighteen times lower than the highest concentration containing the SL-2 sample (1.24 ng-TEQ/teaspoon). TEQs revealed that halogenated PAHs might pose a substantial health risk in Sri Lankan milk powder samples, whereas parent PAHs may pose greater health risks in Japanese samples (Figure 4b). In the case of parent PAHs, JP-4 (1.79 ng-TEQ/teaspoon) had the highest sum of TEQ, followed by JP-3 (0.59 ng-TEQ/teaspoon), and JP-1 (0.41 ng-TEQ/teaspoon). For the ClPAHs, SL-2 (0.95 ng-TEQ/teaspoon) had the highest TEQ, followed by JP-1 (0.30 ng-TEQ/teaspoon) and JP-3 (0.18 ng-TEQ/teaspoon); however, all other samples had TEQ values that were one to two orders lower. Similarly, BrPAHs revealed the highest risk level in the SL-2 (0.28 ng-TEQ/teaspoon) sample, but other samples had TEQ values at least 4–10 fold lower. These results imply that the PAH-like health risk posed by the HPAHs in these milk powders may be increased (Figure 4b). To date, the carcinogenicity of HPAHs has not been adequately addressed. Notwithstanding, the potential hazard of PAHs and HPAHs in milk powder should not be overlooked because they can cause aryl hydrocarbon receptor-mediated genotoxicity as well as being implicated in other toxicological effects [25]. Furthermore, the various REF and TEF values proposed for PAHs and related compounds may underestimate their toxicity [10,26]. It is worth noting that many PAH and HPAH congeners lack TEF or REP values, forcing us to disregard those compounds in the final TEQ estimations. As a result, the derived TEQ values shown here may be understated. In the future, it will be necessary to propose and update TEF and REP values for all detectable PAHs and HPAHs.

## 4. Conclusions

The presence of 75 congeners of PAHs and HPAHs in eight milk powder samples from Sri Lanka and Japan is investigated in this study. In the samples, 53 congeners were detected out of 75. Chlorinated PAHs were the most prevalent in Sri Lankan milk powders, followed by brominated and parent PAHs. Conversely, parent PAH levels were several orders of magnitude higher in Japanese milk powders. Halogenated pyrene, chlorinated fluoranthene, brominated naphthalene, and chlorinated benzo[a]pyrene were almost ubiquitous among the various congeners. The toxic potential estimated by the TEQ method revealed that the contribution of PAHs and HPAHs in milk powder to health risks differed between the two countries. The data suggest that dietary exposure to HPAHs may additively magnify the risks to human health risks. Nonetheless, due to a lack of sufficient TEF or REP values for detected target compounds, the cumulative TEQ values presented here may be underestimated.

## Figures and Tables

**Table 1:** Range or mean level of total parent, chlorinated, and brominated PAHs in milk powders.

Type of Product	Country of Sample	Concentration Ranges or Mean (ng/g Dry Weight)			Method of	Reference
		Sum of Parent PAHs	Sum of ClPAHs	Sum of BrPAHs	Determination	
Milk powder	Argentina and Brazil	11.8–78.4	Not studied	Not studied	HPLC/DAD	[21]
Milk powder	Romania	0.47–1.32	Not studied	Not studied	GC/MS	[23]
Infant formula	Japan	2.01	Not studied	Not studied	HPLC/fluorescence detector	[3]
Milk-based baby food	Italy	52.25	Not studied	Not studied	HPLC/fluorescence detector	[22]
Milk powder	China	2.37–11.83	n.d.–7.38	Not studied	GC-QqQ-MS	[14]
Milk powder	Sri Lanka	n.d.–0.13	0.01–3.35	1.20–5.15	Orbitrap GC/MS	This study
Milk powder	Japan	<0.001–16	0.04–2.54	n.d.–2.03	Orbitrap GC/MS	This study

## Data Availability

All data are presented in the text and in the Supplementary Materials. They are also available on request from the corresponding authors.

## Supplementary Materials

The following supporting information can be downloaded at: https://www.mdpi.com/article/10.3390/toxics10100621/s1, Figure S1. Principal component analysis showing the clustering of different samples from Sri Lanka and Japan with different halogenated PAH and parent PAH congeners. The (A) refers to distribution of different PAH and HPAH compounds in the first two principal components and (B) denotes clustering of different milk powder samples based upon principal scores. Zero concentration (not detected) congeners were not used for analysis. Different numbers denotes different compounds; Table S1. Details of milk powder samples; Table S2: Summary of compounds of interest, their corresponding TEF or REP values, detection frequencies, and concentrations (ng/g d.w.) in milk powder obtained from Sri Lanka and Japan; Table S3. TEQ values calculated from corresponding TEF (for PAHs) or REP (for HPAHs) values for the milk powder samples from Sri Lanka and Japan. Results represented as ng-TEQ/teaspoon of milk powder dry weight.
