# Supplementary material for: Presence of Halogenated Polycyclic Aromatic Hydrocarbons in Milk Powder and the Consequence to Human Health
Source: Toxics. 2022 Oct 19;10(10):621. doi: 10.3390/toxics10100621 (PMC9611289; doi:10.3390/toxics10100621)
Supplement: Supplementary file 1 [file toxics-10-00621-s001.zip › toxics-1952553-supplementary.pdf]

Supplementary information

Presence of Halogenated Polycyclic Aromatic Hydrocarbons in Milk Powder and the Consequence to Human Health

Prasun Goswami [1], Anura Upasanta-Kumara Wickrama-Arachchige [2], Momoka Yamada [3], Takeshi Ohura [3,*] and Keerthi S. Guruge [4,5,*]

1 Atal Centre for Ocean Science and Technology for Islands, ESSO-National Institute of Ocean Technology, Port Blair 744103, Andaman and Nicobar Islands, India
2 Faculty of Fisheries and Ocean Sciences, Ocean University of Sri Lanka, Tangalle HB 82200, Sri Lanka
3 Faculty of Agriculture, Meijo University, Nagoya 468-8502, Japan
4 National Institute of Animal Health, National Agriculture and Food Research Organization, Tsukuba 305-0856, Japan
5 Graduate School of Life and Environmental Sciences, Osaka Metropolitan University, Osaka 598-8531, Japan
* Correspondence: ohura@meijo-u.ac.jp (T.O.); guruge@affrc.go.jp (K.S.G.)

Table S1. Details of milk powder samples

Country	Sample no	Type	fat %
Japan	JP-1	Adult	1.8
	JP-2	Baby formula	0.9
	JP-3	Low fat creaming powder	<0.1
	JP-4	Creaming powder	1.6
Sri Lanka	SL-1	Full cream, imported	13
	SL-2	Full cream, imported	19
	SL-3	Full cream, local	6.2
	SL-4	Full cream, imported	17

Table S2: Summary of compounds of interest, their corresponding TEF or REP values, detection frequencies, and concentrations (ng/g d.w.) in milk powder obtained from Sri Lanka and Japan.

**Congener**	**Acronym**	**# of rings**	**TEF/REP**	**Sri Lanka**			**Japan**		
**PAH**				% of detection	Mean	SD	% of Detection	Mean	SD
Naphthalene	Nap	2	0.001	-	-	-	-	-	-
Acenaphtylene	Acy	3	0.001	-	-	-	-	-	-
Acenaphthene	Ace	3	0.001	-	-	-	50	0.10	0.11
Fluorene	Fl	3	0.001	-	-	-	25	0.19	0.39
Phenanthrene	Phe	3	0.001	-	-	-	50	1.35	2.46
Anthracene	Ant	3	0.01	-	-	-	25	0.32	0.64
Fluoranthene	Fluor	4	0.001	-	-	-	50	2.02	2.95
Pyrene	Py	4	0.001	-	-	-	50	1.89	2.52
Benz[c]phenanthrene	BcPhe	4		-	-	-	50	0.09	0.11
Benz[a]anthracene	BaA	4	0.1	-	-	-	50	0.30	0.37
Chrysene	Chry	4	0.01	-	-	-	50	0.28	0.35
7,12-Dimethylbenz[a]anthracene	7,12-DMBaA	4		25	0.01	0.02	25	1.91×10^{-4}	3.81×10^{-4}
Benzo[b]fluoranthene	BbF	5	0.1	-	-	-	75	0.13	0.18
Benzo[j]fluoranthene + Benzo[k]fluoranthene	BjF+BkF	5	0.1	-	-	-	75	0.06	0.07
Benzo[e]pyrene	BeP	5		-	-	-	75	0.14	0.18
Benzo[a]pyrene	BaP	5	1	-	-	-	75	0.16	0.21

3-Methylcholanthrene	3-MECA	5		25	0.02	0.04	75	1.57×10^{-3}	1.70×10^{-3}
Indeno[1,2,3-cd]pyrene	IP	6	0.1	-	-	-	75	0.21	0.18
Dibenz[a,h]anthracene	DBahA	5	1	-	-	-	75	0.02	0.02
Benzo[g,h,i]perylene	BghiP	6	0.01	-	-	-	75	0.13	0.09
Dibenzo[a,l]pyrene	DBalP	6		50	3.37×10^{-3}	5.75×10^{-3}	50	0.03	0.04
Dibenzo[a,i]pyrene	DBaiP	6		-	-	-	-	-	-
Dibenzo[a,h]pyrene	DBahP	6		-	-	-	-	-	-
**ClPAH**									
9-chlorofluorene	9-ClFlu	3		-	-	-	-	-	-
9-chlorophenanthrene	9-ClPhe	3	0.03	25	0.01	0.03	25	2.09×10^{-3}	4.18×10^{-3}
2-chloroanthracene + 1-chloroanthracene	2-ClAnt+1-ClAnt	3	0.1	50	5.89×10^{-4}	9.15×10^{-4}	50	1.96×10^{-3}	2.57×10^{-3}
9-chloroanthracene	9-ClAnt	3	0.03	-	-	-	50	4.71×10^{-3}	7.05×10^{-3}
3,9-dichlorophenanthrene	3,9-Cl2Phe	3	0.32	50	2.59×10^{-3}	4.14×10^{-3}	75	1.21×10^{-3}	1.41×10^{-3}
9,10-dichloroanthracene + 1,9-dichlorophenanthrene	9,10-Cl2Ant +1,9-Cl2Phe	3	0.2	100	4.97×10^{-3}	4.01×10^{-3}	75	5.79×10^{-4}	7.32×10^{-4}
9,10-dichlorophenanthrene	9,10-Cl2Phe	3	0.16	25	5.51×10^{-3}	1.10×10^{-2}	50	2.02×10^{-3}	2.47×10^{-3}

3-chlorofluoranthene	3-ClFluor	4	0.17	-	-	-	25	2.11×10^{-3}	4.21×10^{-3}
8-chlorofluoranthene	8-ClFluor	4	0.18	25	1.79×10^{-5}	3.57×10^{-5}	75	5.53×10^{-4}	5.98×10^{-4}
1-chloropyrene	1-ClPy	4	0.1	100	0.41	0.65	50	0.14	0.17
3,9,10-trichlorophenanthrene	3,9,10-Cl3Phe	3	0.77	75	8.74×10^{-4}	1.11×10^{-3}	-	-	-
1,3-dichlorofluoranthene	1,3-Cl2Fluor	4		50	4.61×10^{-3}	8.58×10^{-3}	50	1.23×10^{-3}	2.16×10^{-3}
3,8-dichlorofluoranthene	3,8-Cl2Fluor	4	5.7	25	9.44×10^{-3}	1.89×10^{-2}	50	0.01	0.01
3,6,9-trichlorophenanthrene	3,6,9-Cl3Phe	3		-	-	-	-	-	-
Dichloropyrene	Cl2Py	5		75	0.14	0.17	50	0.06	0.10
3,4-dichlorofluoranthene	3,4-Cl2Fluor	4		75	0.25	0.35	50	0.07	0.11
6-chlorochrysene	6-ClChry	4	2.1	-	-	-	-	-	-
7-chlorobenz[a]anthracene	7-ClBaA	4	0.83	75	7.82×10^{-4}	5.71×10^{-4}	50	8.57×10^{-4}	1.28×10^{-3}
Trichloropyrene	Cl3Py	5		25	0.03	0.06	50	0.04	0.05
6,12-dichlorochrysene	6,12-Cl2Chry	4	0.03	-	-	-	-	-	-
7,12-dichlorobenz[a]anthracene	7,12-Cl2BaA	4	0.1	-	-	-	-	-	-
Tetrachloropyrene	Cl4Py	4		25	0.06	0.11	75	0.12	0.11
Tetrachlorofluoranthene	Cl4Fluor	4		-	-	-	50	0.01	0.01

6-chlorobenzo[a]pyrene	6-ClBaP	5	0.09	25	8.75×10^{-4}	1.75×10^{-3}	100	0.02	0.01
Chloroperylene	ClPery	5		-	-	-	-	-	-
Dichlorobenzo[a]pyrene	Cl2BaP	5		25	0.07	0.15	75	0.59	0.66
Dichloroperylene	Cl2Pery	5		-	-	-	-	-	-
Trichlorobenzo[a]pyrene	Cl3BaP	5		-	-	-	50	0.06	0.07
**BrPAH**									
2-Bromonaphthalene	2-BrNap	2		100	0.54	0.19	25	3.03×10^{-4}	6.07×10^{-4}
α-Bromonaphthalene	α-BrNap	2		100	1.04	0.49	25	7.50×10^{-4}	1.50×10^{-3}
1,4-Dibromonaphthalene	1,4-Br2Nap	2		50	6.24×10^{-4}	7.49×10^{-4}	-	-	-
2,7-Dibromonaphthalene	2,7-Br2Nap	2		-	-	-	-	-	-
9-Bromofluorene	9-BrFlu	3		-	-	-	-	-	-
2-Bromofluorene	2-BrFlu	3	0.02	25	1.25×10^{-4}	2.50×10^{-4}	-	-	-
9-Bromophenanthrene	9-BrPhe	3	0.02	25	2.50×10^{-4}	5.00×10^{-4}	-	-	-
9-Bromoanthracene	9-BrAnt	3	0.01	-	-	-	-	-	-
Bromofluoranthene	BrFluor	4		-	-	-	-	-	-
9,10-Dibromoanthracene	9,10-Br2Ant	3		25	2.50×10^{-4}	5.00×10^{-4}	-	-	-
1-Bromopyrene	1-BrPy	4	0.04	75	0.80	1.30	50	0.25	0.33
7-Bromobenz[a]anthracene	7-BrBaA	4	0.84	-	-	-	-	-	-

Dibromopyrene	Br2Py	4		50	0.21	0.35	50	0.76	0.89
7,11-Dibromobenz[a]anthracene	7,11-Br2BaA	4	0.06	-	-	-	-	-	-
7,12-Dibromobenz[a]anthracene	7,12-Br2BaA	4	0.09	-	-	-	-	-	-
4,7-Dibromobenz[a]anthracene	4,7-Br2BaA	4	0.77	-	-	-	25	1.25×10^{-4}	2.50×10^{-4}
5,7-Dibromobenz[a]anthracene	5,7-Br2BaA	4	0.02	-	-	-	25	1.25×10^{-4}	2.50×10^{-4}
6-Bromobenzo[a]pyrene	6-BrBaP	5	0.002	-	-	-	-	-	-
Tetrabromopyrene	Br4Py	4		-	-	-	-	-	-
Dibromobenzo[a]pyrene	Br2BaP	5		-	-	-	-	-	-
Tribromobenzo[a]pyrene	Br3BaP	5		-	-	-	-	-	-

SD: standard deviation

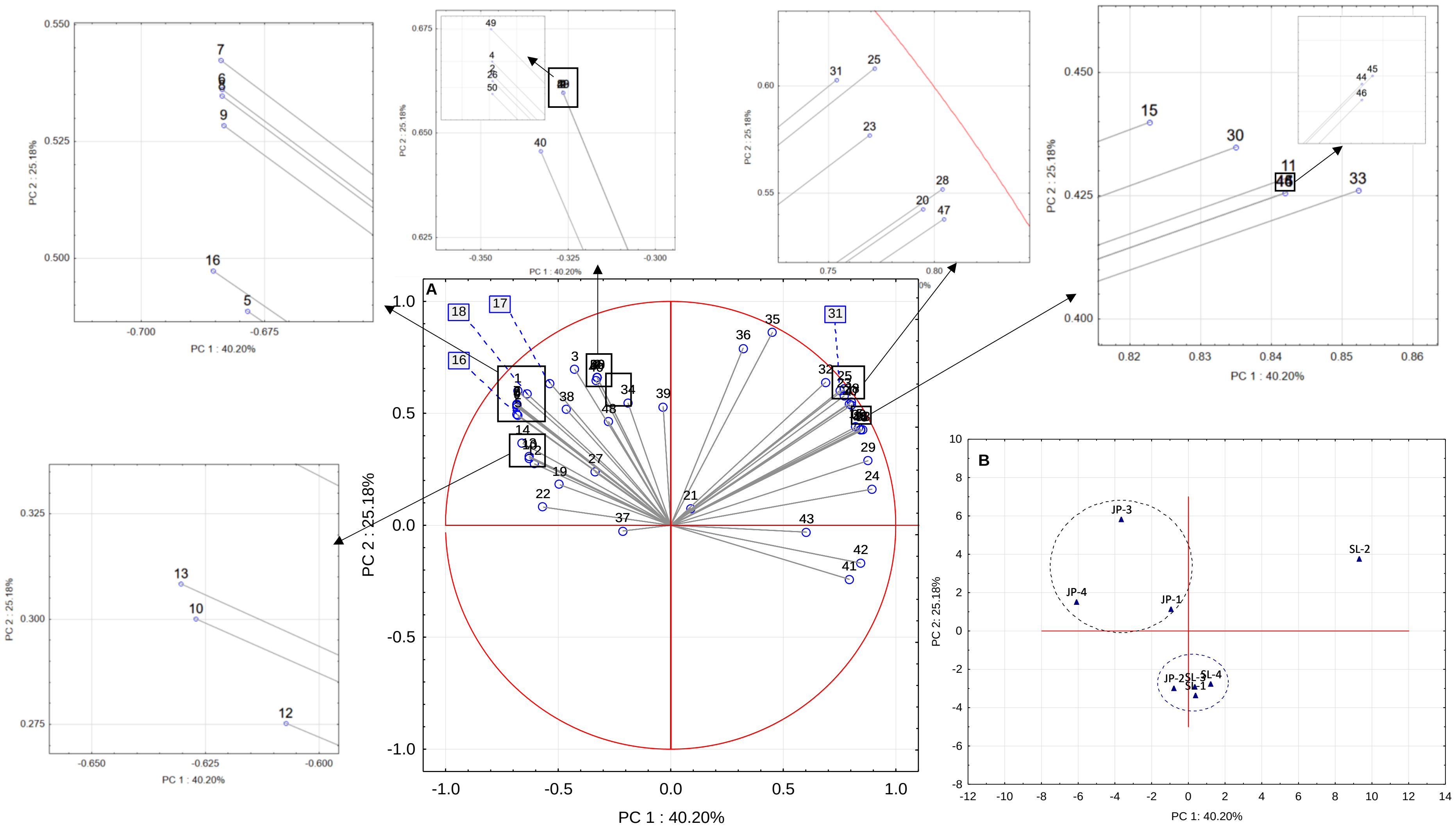

Figure S1. Principal component analysis showing the clustering of different samples from Sri Lanka and Japan with different halogenated PAH and parent PAH congeners. The (A) refers to distribution of different PAH and HPAH compounds in the first

two principal components and (B) denotes clustering of different milk powder samples based upon principal scores. Zero concentration (not detected) congeners were not used for analysis. Different numbers denotes different compounds. Where, 1 – Ace; 2–Flu; 3 – Phe; 4 – Ant; 5 – Fluor; 6 – Py; 7 – BcPhe; 8 – BaA; 9 – Chry; 10 - BbF+BjF; 11 - 7,12-DMBaA; 12 – BkF; 13 – BeP; 14 – BaP; 15 - 3-MECA; 16 – IDP; 17 – DbahA; 18 – BghiP; 19 – DbalP; 20 - 9-ClPhe; 21 - 1-ClAnt+2-ClAnt; 22 - 9-ClAnt; 23 - 3,9-Cl2Phe; 24 - 9,10-Cl2Ant+1,9-Cl2Phe; 25 - 9,10-Cl2Phe; 26 - 3-ClFluor; 27 - 8-ClFluor; 28 - 1-ClPy; 29 - 3,9,10-Cl3Phe; 30 - 1,3-Cl2Fluor; 31 - 3,8-Cl2Fluor; 32 - Cl2Py; 33 - 3,4-Cl2Fluor; 34 - 7-ClBaA; 35 - Cl3Py; 36 - Cl4Py; 37 - Cl4Fluor; 38 - 6-ClBaP; 39 - Cl2Bap; 40 - Cl3Bap; 41 - 2-BrNap; 42 - α-BrNap; 43 - 1,4-Br2Nap; 44 - 2-BrFlu; 45 - 9-BrPhe; 46 - 9,10-Br2Ant; 47 - 1-BrPy; 48 - Br2Py; 49 - 4,7-Br2BaA; 50 - 5,7-Br2BaA.

Table S3. TEQ values calculated from corresponding TEF (for PAHs) or REP (for HPAHs) values for the milk powder samples from Sri Lanka and Japan. Results represented as ng-TEQ/teaspoon of milk powder dry weight.

		Sri Lanka				Japan			
Congeners	TEF or REP	SL-1	SL-2	SL-3	SL-4	JP-1	JP-2	JP-3	JP-4
Ace	0.001	0	0	0	0	0	0	0.00045	0.000547
Fl	0.001	0	0	0	0	0	0	0.002019	0
Phe	0.001	0	0	0	0	0	0	0.013092	0.000953
Ant	0.01	0	0	0	0	0	0	0.03303	0
Fluor	0.001	0	0	0	0	0	0	0.004779	0.016269
Py	0.001	0	0	0	0	0	0	0.005856	0.01381
BaA	0.1	0	0	0	0	0	0	0.113113	0.198627
Chry	0.01	0	0	0	0	0	0	0.01052	0.01897
BbF	0.1	0	0	0	0	0.017072	0	0.012967	0.101856
BjF+BkF	0.1	0	0	0	0	0.014407	0	0.003542	0.042777
BaP	1	0	0	0	0	0.216629	0	0.257125	1.222108

IP	0.1	0	0	0	0	0.061853	0	0.045743	0.114693
DBahA	1	0	0	0	0	0.09707	0	0.080304	0.051289
BghiP	0.01	0	0	0	0	0.005152	0	0.003714	0.004872
9-ClPhe	0.03	0	0.003928	0	0	0	0	0.000651	0
1-ClAnt+2-ClAnt	0.1	0.000111	0.000501	0	0	0	0.001407	0.000631	0
9-ClAnt	0.03	0	0	0	0	0	0.000306	0	0.001163
3,9-Cl2Phe	0.32	0.001396	0.00722	0	0	0.002224	0	0.001812	0
9,10-Cl2Ant + 1,9-Cl2Phe	0.2	0.000576	0.005255	0.001343	0.003169	0.000834	5.56×10^{-5}	0.000316	0
9,10-Cl2Phe	0.16	0	0.009166	0	0	0.00126	0	0.002094	0
3-ClFluor5	0.17	0	0	0	0	0	0	0.003723	0
8-ClFluor	0.18	3.34×10^{-5}	0	0	0	0	0.0005	0.000501	3.34×10^{-5}
1-ClPy	0.1	0.001226	0.359358	0.038807	0.023668	0.090346	0	0.053067	0
3,9,10-Cl3Phe	0.77	0.001001	0.005005	0.000991	0	0	0	0	0
3,8-Cl2Fluor	5.7	0	0.559427	0	0	0.195953	0	0.107386	0
7-ClBaA	0.83	0	0.001541	0.002595	0.002615	0	0	0.005854	0.00154
6-ClBaP	0.09	0	0.000819	0	0	0.007706	0.00035	0.003274	0.005025
2-BrFl	0.02	0	2.6×10^{-5}	0	0	0	0	0	0
9-BrPhe	0.02	0	5.2×10^{-5}	0	0	0	0	0	0
1-BrPy	0.04	0	0.28434	0.02139	0.026226	0.072095	0	0.032864	0
4,7-Br2BaA	0.77	0	0	0	0	0	0	0.001	0
5,7-Br2BaA	0.02	0	0	0	0	0	0	2.6×10^{-5}	0

Weight of milk powder in a teaspoon = 2.6 g
